# Expanding the Role of Early Health Economic Modelling in Evaluation of Health Technologies Comment on "Problems and Promises of Health Technologies: The Role of Early Health Economic Modeling"

**DOI:** 10.15171/ijhpm.2020.18

**Published:** 2020-02-05

**Authors:** Carlo Federici, Aleksandra Torbica

**Affiliations:** ^1^Centre for Research on Health and Social Care Management (CERGAS), SDA Bocconi School of Management, Bocconi University, Milan, Italy.; ^2^University of Warwick, School of Engineering, Coventry, UK.

**Keywords:** Early Economic Modelling, Health Technology Assessment, Cost-Effectiveness, Early Dialogue

## Abstract

In this commentary, we discuss early stage assessments of innovative medical technologies both in terms of methods applied as well as their use in healthcare decision-making. We argue that cost-effectiveness alone may be too reductive if taken as the only decision rule, and it would benefit from being used within a broader evaluation framework. We discuss innovative methods which may contribute to better estimate the potential costs and consequences of a technology in the absence of solid clinical data, as frequently the case in early assessments. Finally, we comment on the potential synergies which may take place should early economic models be used not only by technology developers alone but as a negotiating base during early dialogues with health technology assessment (HTA) bodies.

## Introduction

The concept of early-stage health economic modelling has been around since the mid-nineties,^1-4^ and its potential role to support technology developers decisions have been broadly explored in a number of reviews.^5-8^ For example Hartz and colleagues argue that early health economic modelling may support technology developers by providing relevant insights on strategic R&D decision-making, pre-clinical preliminary market assessments, go/no-go decisions and identification of potentially successful projects, development of future trial design, assessment of future reimbursement and pricing scenarios and price determination.^6^

The work by Grutters et al,^9^ recently published in *IJHPM*, nicely reports on real case studies providing interesting insights on how early health economic models have been actually implemented and how they have been used to assess the potential cost-effectiveness and inform further development, implementation and positioning of innovations in clinical practice. The authors disclose and discuss data on previously developed early health economic modelling assessments on 30 innovations conducted by their team, all of which were non-drug technologies.

While commenting on the article by Grutters et al, we discuss some general aspects to be considered when conducting an early economic model and more generally early stage assessments of innovative medical technologies. First, we argue that, at least from the developers’ perspective, cost-effectiveness alone may be too reductive if taken as the only decision rule, and it would benefit from being used within a broader evaluation framework. Second, we discuss the potential to explore further methods which may contribute to better estimate the potential costs and consequences of a technology in the absence of solid clinical data. Finally, we discuss the potential synergies which may take place should early economic models be used not only by technology developers alone but as a negotiating base during early dialogues with health technology assessment (HTA) bodies.

## Cost-Effectiveness as the Only Decision Rule

In many jurisdictions, evidence on cost-effectiveness plays a fundamental role in the decision process on coverage and reimbursement, due to its role in informing optimal allocative efficiency of limited healthcare budgets. Therefore, early assessments of health technologies cannot prescind from this type of analyses. However, as the authors explicitly argue in the discussion, using cost-effectiveness as the only decision rule may be an over-simplification of reality, as commercial viability or the potential value of a technology may be affected by other dimensions within the HTA spectrum such as the expected budget impact, the ability to meet the needs of patients or clinicians, market dynamics and competing upcoming innovations as well as organizational and logistical issues.^10^ For example, clinicians may be more interested in the capacity of the technology to achieve a certain minimum clinical difference rather than its potential cost-effectiveness. Similarly, those in charge of commissioning technologies may be willing to purchase innovations only if they do not compromise short-term financial sustainability, in rigid budget structures defined by silos. Therefore, from the perspective of the technology developers, these aspects could be equally important criteria to inform decisions regarding further technology development as they may directly impact future return on investments and revenues. Therefore, one may argue that any early-stage assessment of a technology should consider as closely as possible all the major aspects that will affect not only coverage and reimbursement, but also adoption and diffusion. The identification of these drivers is likely to be technology and setting specific and span across several different stakeholders. For example, in many countries, medical devices do not go through formal HTA processes at the national level, so that, once the European Conformity (CE) mark has been granted, commissioning decisions take place at a more local level, based on criteria that are likely to be different from or not exclusively based on cost-effectiveness.

Some methods to quantitatively incorporate other dimensions besides cost-effectiveness have been proposed in the literature.^11^ For example Retèl et al^12^ used scenario drafting or road-mapping, including patients and organizational aspects. Cosh et al^13^ included headroom analysis based on an early cost-effectiveness analysis into a broader decision framework which comprehend other assessments such as strategic considerations, clinical problem definition, return on investment analysis and further economic analysis. Åstebro and Elhedhli used simple decision heuristic to derive an estimate for the likelihood that early-stage ventures are subsequently commercialized.^14^

It is worth noting that in their assessments, the authors did not identify any technology for which a firm no-go decision was recommended. This is an interesting result as early health economic modelling has been often claimed to be able to support technology developers in taking this type of strategic go/no-go decisions.^6,8^ However, this may be the result of the assumption that the innovations under assessment will perform at least as well as the comparator used in the analysis, so that only the II and III quadrant of the cost-effectiveness plane were likely considered. In this case, in theory it is always possible to find combination of price/effects that would make the technology cost-effective. It is not clear whether the authors in their assessments also considered a lower minimum acceptable price of the technology below which technology developers would consider unlikely to have a satisfactory return on investments. Again, insights on the potential cost-effectiveness of the technology would be more valuable if coupled to other types of assessments which consider the ultimate objective of technology developers to make a profit out of their developed products.

## Expanding Methods to Forecast Technology Performance

As clearly argued by the authors, headroom analyses represent best case and often unrealistic scenarios. While the use of this simple techniques is made necessary by the lack of clinical and economic data on the innovation, it is worth considering whether more could be done to better define credibility ranges regarding the performance of innovative medical technologies, thus improving the output of early economic evaluations. Certainly, this task is not trivial, and it is highly dependent on the characteristics of the innovation and its intended mechanism of action through which better health outcomes are expected to be generated. For example predicting the performance of an e-health app which is intended to improve adherence to treatment, by collecting patient-reported outcomes and allowing better communication between oncologic patients and clinicians is going to be very different to predicting the safety and effectiveness profiles of an innovative total artificial heart for patients with advanced heart failure. For example, insights for the former could be drawn from health psychology theories^15^ or by using preference elicitation techniques such as discrete choice experiments^16^ to identify factors which affect adherence, whereas the latter could exploit the use of computer modelling and simulations to predict possible technology failure modes, explore potential heterogeneity in the patient population and provide credible ranges of the effects of the technology on intermediate or final clinical endpoints. Hartz and John, and Miller et al propose the use of computer model simulations to predict the outcomes of a clinical trial (Clinical trial simulation – CTS).^6,7^ CTS uses synthetic mathematical models to integrate different sub-systems that determine the mechanisms of action of a technology and its way of interacting with the environment. For example, these simulations use pharmacodynamics and pharmacokinetics models, biomechanics and other mechanistic models, together with epidemiological data on disease progression, patients’ heterogeneity in response to treatment, compliance, learning curves etc to simulate the results on clinically relevant endpoints in a target population. Results from CTS models can then be used to populate early economic evaluations that will be subsequently updated if new evidence from in vivo clinical trials becomes available. For example, Bhattacharya et al developed a multiscale model to predict the absolute probability of a fracture following a fall. The model combined three mechanistic sub-models at different scales which modelled the impact force on the body applied by the floor during a fall; the fraction of impact force transferred to the skeleton; and the subjects’ bone strength.^17^ By using a virtual cohort of subjects with specific features including body mass, height, geometry and elastic properties of the proximal femur, the model could predict the relative effectiveness of different interventions aiming at preventing hip fractures, such as for example hip protector devices.

In addition to serving as a preliminary source of evidence for early cost-effectiveness models, CTS models can improve the efficiency of clinical development pathways. Particularly, CTS can inform better study protocols, including the choice of the endpoints that are relevant to different stakeholders, and calculation of the most appropriate sample size, to maximize the probability of obtaining statistically significant estimates. These methods have raised increasing interest among regulators, both in the United States and Europe^18,19^ but their use for HTA purposes is still broadly unexplored.

## Early Health Economic Modelling Within the Context of Early Dialogues With HTA Bodies

Traditionally HTA has been conducted at a point in time at which technologies are close to their final design and pivotal clinical evidence has been already generated. By this time developers have sustained relevant unrecoverable investment costs and payers have no longer the possibility to influence product development, and are often compelled to bare the risk of taking uncertain decisions with partial and/or unsatisfactory evidence. As the authors clearly showed, early health economic modelling can provide useful recommendations on how innovations should be further developed or implemented to enhance cost-effectiveness, including identifying the optimal indication and positioning of the innovation in the care pathway. They also showed its potential to inform the need for further research by identifying the parameters which are most likely to affect the overall uncertainty on the technology’s cost-effectiveness profile. We argue that, while valuable *per se* to technology developers, this information could be even more valuable if used within the context of early dialogues with payers and HTA bodies at early stages of product development.

For example, when used within established national or European early dialogue process, early economic models could be used as the main guiding analysis to define the protocol of the pivotal clinical trial and the questions which technology developers may ask to HTA bodies about their intended clinical and economic evidence generation plan. While the authors mainly used deterministic sensitivity analysis, this task could be performed using more advanced techniques such as value of information analysis (VOI) tools.^20^ VOI exploits economic models and the outputs of probabilistic sensitivity analyses (PSA) to estimate the potential value of conducting further research to resolve the existing uncertainty around a technology. Yet, the authors argue that PSA, on which VOI is based, could be misleading at this early stage, as many uncertainties affecting the estimation of the costs or consequences of the intervention would be hard to quantify, ultimately causing pseudo-certainty. While we agree on the need to combine scenario, as well as deterministic and PSA to explore all potential sources of uncertainty and their interrelatedness, we believe that even at this early stage PSA is the most appropriate way to characterize (statistical) parameter uncertainty, and that decisions on further product development should be based on what is actually known at the time of the assessment. VOI techniques have developed considerably in the last years covering not only the identification of model parameters for which further research would be valuable, but also key of aspects related to the design of future research, including optimal target population, study design, duration, sample sizes, or the optimal combination of sequential studies in a clinical development plan. In principle, VOI analyses could also help defining what needs to be collected before market access, and what can be collected afterwards through post-market clinical follow up study or more formally agreed Coverage with evidence development schemes. However, again, it should be clear that by using early economic modelling and VOI to specify the clinical development of a technology, cost-effectiveness would be the only guiding criteria used to take decisions, which may not be in line with what matters the most to HTA bodies in many European countries. More research is needed to understand the acceptability of these methods and their underlying principles by decision-makers across Europe, as well as their applicability within the context of early dialogues.

In conclusion we would like to underline that, while there has been an increasing interest in early health economic modelling and their role in the governance of innovation in healthcare, very few empirical applications are publicly available. We very much welcome studies like the one by Grutters and colleagues as they fill this important gap in the literature on an increasingly important topic from both an academic as well as a policy perspective.

## Ethical issues

Not applicable.

## Competing interests

Authors declare that they have no competing interests.

## Authors’ contributions

Both authors have made substantial contributions to the conception and design of the manuscript and have been involved in drafting it as well as revising it critically. All authors have given final approval of the version to be published.

## Authors’ affilaitions

^1^Centre for Research on Health and Social Care Management (CERGAS), SDA Bocconi School of Management, Bocconi University, Milan, Italy. ^1^University of Warwick, School of Engineering, Coventry, UK.
